# HIV Tat Expression and Cocaine Exposure Lead to Sex- and Age-Specific Changes of the Microbiota Composition in the Gut

**DOI:** 10.3390/microorganisms11030799

**Published:** 2023-03-21

**Authors:** Lu Li, Xiaojie Zhao, Johnny J. He

**Affiliations:** 1 Department of Microbiology and Immunology, Chicago Medical School, Rosalind Franklin University, 3333 Green Bay Road, North Chicago, IL 60064, USA; 2 Center for Cancer Cell Biology, Immunology and Infection, Rosalind Franklin University, North Chicago, IL 60064, USA; 3 School of Graduate and Postdoctoral Studies, Rosalind Franklin University, North Chicago, IL 60064, USA

**Keywords:** HIV Tat, cocaine, gut microbiota, brain, 16S rRNA gene sequencing

## Abstract

The balance of microbial communities in the gut is extremely important for normal physiological function. Disruption of the balance is often associated with various disorders and diseases. Both HIV infection and cocaine use are known to change the gut microbiota and the epithelial barrier integrity, which contribute to inflammation and immune activation. Our recent study shows that Tat expression and cocaine exposure result in changes of genome-wide DNA methylation and gene expression and lead to worsen the learning and memory impairments. In the current study, we extended the study to determine effects of Tat and cocaine on the gut microbiota composition. We found that both Tat expression and cocaine exposure increased Alteromonadaceae in 6-month-old female/male mice. In addition, we found that Tat, cocaine, or both increased Alteromonadaceae, Bacteroidaceae, Cyanobiaceae, Erysipelotrichaceae, and Muribaculaceae but decreased Clostridiales_vadinBB60_group, Desulfovibrionaceae, Helicobacteraceae, Lachnospiraceae, and Ruminococcaceae in 12-month-old female mice. Lastly, we analyzed changes of metabolic pathways and found that Tat decreased energy metabolism and nucleotide metabolism, and increased lipid metabolism and metabolism of other amino acids while cocaine increased lipid metabolism in 12-month-old female mice. These results demonstrated that Tat expression and cocaine exposure resulted in significant changes of the gut microbiota in an age- and sex-dependent manner and provide additional evidence to support the bidirectional gut–brain axis hypothesis.

## 1. Introduction

Up to 100 trillion (10^14^) microbes colonize in human gut and are collectively called gut microbiota [1,2]. There are about 2200 species and 12 phyla, which mostly fall into four phyla: Protebacteria, Firmicutes, Actinobacteria, and Bacteroidetes [3,4]. They are believed to originate at birth and evolve over the course of biological ageing [5,6,7,8,9]. Host genetics, environment, and lifestyle contribute to the changes of their composition [4,10,11]. The dynamic crosstalk between these microbes and the host also affects the host’s health [1,10]. The balance of various microbial communities in the gut plays a fundamental role in normal physiological functions such as breakdown of food for absorption, protection against pathogens, elicitation of immunity, and maintenance of the barrier integrity of the gut and intestines [12,13,14]. Thus, disruption of the balance, also known as dysbiosis, is often associated with various disorders and diseases such as cancer, viral infection, and abnormal behaviors [2,15,16,17,18,19]. Microbes, their metabolites such as short-chain fatty acids, their structural components lipopolysaccharides (LPS), and hormones released by gut epithelial enteroendocrine cells can all function to mediate the interactions between the microbiota and the host [2,20].

Early HIV infection and replication in gut-associated lymphoid tissues result in massive CD4 T cell depletion and compromised epithelial barrier and gut immunity and as a result, direct translocation of gut microbes and their products such as LPS into the circulatory system and subsequent systemic inflammation and immune activation [21,22,23,24,25]. HIV infection is also associated with decreased abundance, composition, and diversity of gut microbiota. These changes in turn contribute to persistent inflammation and microbial translocation, dysfunctional metabolism of the host, and HIV disease progression and reservoir size [26,27,28,29,30]. Importantly, antiretroviral therapy does not fully restore the integrity of the gut epithelial barrier and the disrupted gut microbiota that result from HIV infection despite its potent suppressive effects on HIV replication [27,31]. HIV infection, antiretroviral therapy, and changes in gut microbiota have all been linked to HIV comorbidities including metabolic, cardiovascular, and neurocognitive disorders [32,33,34,35,36,37].

In the era of antiretroviral therapy, mild cognitive and motor disorder has become the most common clinical manifestation of HIV-associated neurocognitive disorders (HAND), characterized by persistent neuroinflammation [38,39,40]. HIV viral protein Tat is a major pathogenic factor for HAND and neuroHIV, as its expression in the brain of doxycycline (Dox)-inducible astrocyte-specific HIV Tat transgenic mice (iTat) in the absence of HIV infection leads to locomotor, learning and memory deficits [41,42,43,44,45,46,47,48,49,50,51,52], and astrocyte/microglia activation, chronic neuroinflammation and loss of neuronal integrity [43,48,50,51,53], the consistent neurological and neuropathological hallmarks of HAND and neuroHIV in the era of antiretroviral therapy. Tat is present in the brain of HIV-infected people who are under active antiretroviral therapy [54,55,56]. Several studies suggest gut microbiota as a potential source of the persistent neuroinflammation. Specific gut microbiota signatures, elevated LPS, and systemic immune activation are associated with the severity of HAND [36,57,58], while supplementation of gut microbiota with probiotics decreases neuroinflammation and improves neurocognitive function in the context of HIV infection [59,60]. Use/abuse of substances such as cocaine, methamphetamine, and opioids constitute a major risk factor for HAND [61,62,63], while it alters the gut microbiota and permeability and increase inflammation [64,65,66,67].

In this study, we took advantage of iTat mice, exposed them to cocaine, and determined the changes of the gut microbiota in response to Tat expression and chronic cocaine exposure. Specifically, we harvested large intestines of the mice, extracted the DNA from the tissues, constructed 16S rRNA gene libraries, performed the metagenomic sequencing, and determined the abundance, composition, and diversity of the gut microbiota. Mice of two different ages, 6- and 12 months old, were included in the study. All the data were stratified by genotypes (WT/iTat), treatment (saline/cocaine, SA/CA), ages (6/12 months), and sexes (male/female).

## 2. Materials and Methods

**Experimental design and animals.** Wild-type C57BL/6 mice (Jackson Laboratory, Bar Harbor, ME, USA) and Dox-inducible astrocyte-specific HIV-1 Tat transgenic mice (iTat) were derived from our previous study [43,63]. Animals were fed with Dox-containing diet (0.625 g Dox/kg, Envigo, Indianapolis, IN, USA) for 5 or 11 months from day 21 when they were weaned and continued on the same diet throughout the remaining studies. These animals were given cocaine (CA, i.p. 30 mg/kg/day) or the solvent phosphate-buffered saline (SA) for 14 days, kept drug-free for 10 days, assessed for various behaviors for 20 days, and euthanized to harvest large intestinal tissues. There were a total of 16 experimental groups: 2 mouse strains (WT/iTat) × 2 mouse ages (6/12 months) × 2 sexes (M/F) × 2 treatments (CA/SA) and a total of 192 mice (16 groups × 12 mice/group). All the animal procedures were approved by the Institutional Animal Care and Use Committee.

**DNA extraction.** Large intestine tissues (near anus) were harvested and used to extract DNA using a DNeasy Blood & Tissue Kit (Qiagen, Germantown, MD, USA) according to the manufacturer’s instructions. Briefly, large intestine tissues (about 15 mg) were cut into small pieces, placed into a 1.5 mL microcentrifuge tube containing 180 µL ATL buffer, and treated with 600 mAU/mL proteinase K at 56 °C for 2 hr. When the tissues were completely lysed, DNA was precipitated with 200 µL buffer AL and 200 µL 100% ethanol, washed twice (buffer AW1 and AW2, once each), eluted from the mini-spin column with 200 µL buffer AE, diluted to 10 ng/µL, and stored at −20 °C for subsequent PCR amplification. All DNA preps had the A260/A280 ratio of higher than 1.80.

**16S rRNA gene metagenomic library construction, sequencing, and initial sequence analysis.** The purified DNA was used to construct 16S rRNA gene libraries as previously described [68]. Briefly, the DNA was amplified for the V4 variable region of the bacterial 16S rRNA gene using fusion primers with partial Illumina adaptors. The universal bacterial primers used were 515F: 5′-GTG CCA GCM GCC GCG GTA A-3′ and 806R: 5′-GGA CTA CHV GGG TWT CTA AT-3′. The PCR reaction (25 µL) consisted of 10 ng the purified DNA, 1X AccuStart II PCR Supermix containing Taq DNA polymerase (Quantabio, Beverly, MA), 10 µg BSA, and 500 nM each primer, with a program of 1 cycle of 94 °C for 3 min, 30 cycles of 94 °C for 30 s, 50 °C for 30 s, and 72 °C for 1 min, and a final cycle of 72 °C for 10 min. Amplicon DNA were cleaned, indexed, normalized, and pooled using a MiSeq Reagent kit (Illumina, San Diego, CA, USA) and sequenced on the Illumina MiSeq platform (Illumina, San Diego, CA). The raw reads were processed and paired-end reads (forward and reverse reads) were merged and denoised using the DADA2 algorithm (ver. 1.3.3) [69]. The processed reads datasets from 123 samples (6–11 samples/group) were clustered to operational taxonomic units (OTU) using a similarity threshold of 97% or higher (2,974,646 matched reads) and the taxonomy was assigned using the SILVA reference database (ver. 1.3.2) [70]. The sequencing and the initial sequence analysis were performed by the Center of Bioinformatics and Functional Genomics of Miami University, Oxford, OH.

**Subsequent sequence and statistical analysis.** All OTU data were analyzed by an online analysis tool MicrobiomeAnalyst (ver. 1.0) [71]. The relative abundance (%) was calculated at the family level. The Shannon index was calculated to determine the α diversity and was compared using the Kruskal–Wallis test. The PCoA-Bray–Curtis index was calculated to determine the β diversity and was compared using permutational multivariate analysis of variance (PERMANOVA). The univariate analysis was performed using the Kruskal–Wallis test to compare the specific families among four experimental groups (WT/iTat × SA/CA). The software STAMP (ver. 2.1.3) was used to perform post hoc Games–Howell’s test to compare among multiple groups [72]. “*”, “#”, “&”, “+” and “@” denote *p * <  0.05. The Tax4Fun was used to estimate microbial metabolic functions based on the Kyoto Encyclopedia of Genes and Genomes (KEGG) database [71,73], and the functional metabolic pathways were compared using the Kruskal–Wallis test with a post hoc Games–Howell’s test.

## 3. Results

We analyzed all the sequence reads and identified the 22 most reliable families of gut microbiota as the core microbiota to determine the abundance, and α diversity using the Shannon index which is a quantitative indicator of the number of different families, and β diversity using the PCoA-Bray–Curtis index which quantifies the variability in community composition among samples in mice of different ages and sexes in response to Tat expression and cocaine exposure. Then, we further compared 12 families which were found to be affected in either group for their abundance in mice of different ages and sexes in response to Tat expression and cocaine exposure.

**Figure 1:**
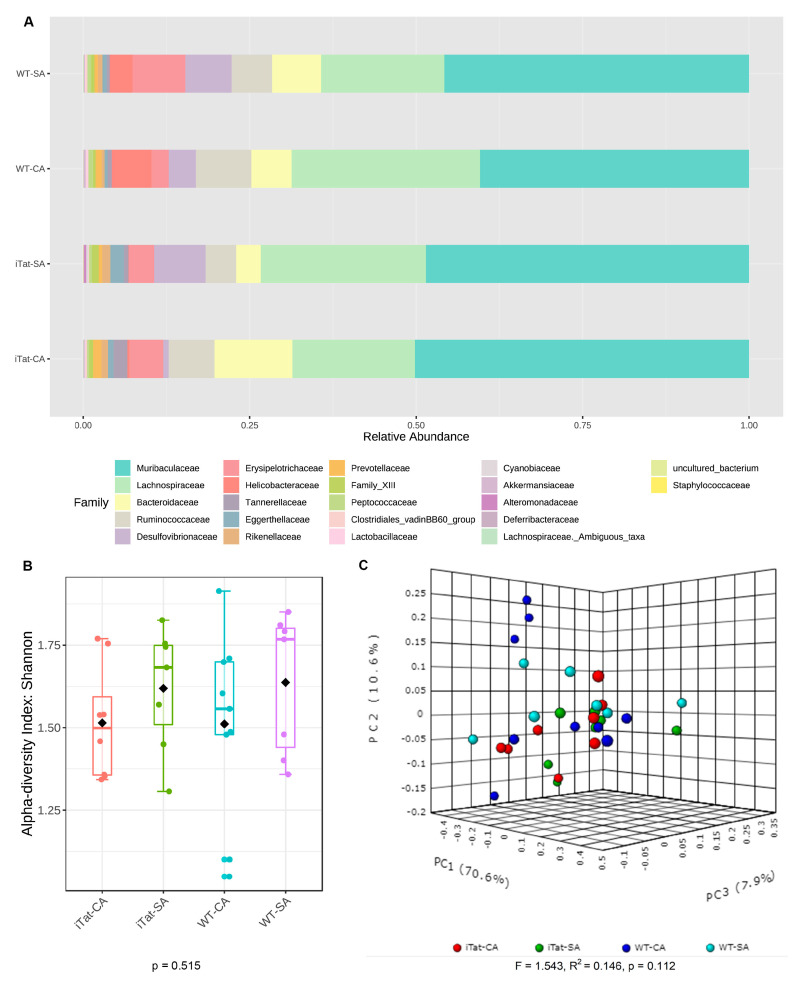
Effects of Tat expression and cocaine exposure on microbial abundance and diversity in the gut of 6-month female mice. (**A**). Relative abundance (%) of top 22 families of gut microbiota in four experimental groups WT-SA, WT-CA. iTat-SA, and iTat-CA were presented as a stacked bar plot. (**B**). Shannon index was calculated to determine α-diversity of the gut microbiota in four experimental groups WT-SA, WT-CA. iTat-SA, and iTat-CA, presented as a box plot, and analyzed using the Kruskal–Wallis test. The error bars were standard derivation (SD). (**C**). Bray–Curtis dissimilarity was calculated to determine β-diversity, presented as a PCoA plot, and analyzed using PERMANOVA. The percentage variation in the plotted principal component were indicated on the axes.

**Figure 2:**
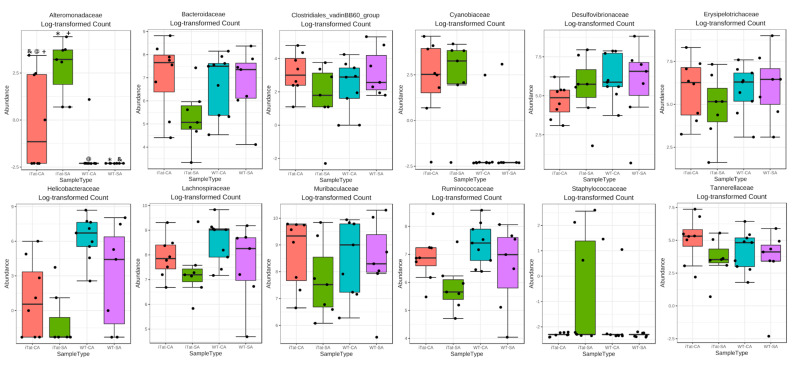
Effects of Tat expression and cocaine exposure on microbial abundance by family in the gut of 6-month female mice. Twelve families which were affected in either group were presented. The data were analyzed using Kruskal–Wallis test, followed by post hoc Games–Howell’s test. The family which was affected by Tat expression and/or cocaine exposure was marked in red. The error bars were SD, and “*”, “&”, “@” or “+” denoted *p* < 0.05.

We first determined the effects of Tat and cocaine on the gut microbiota of 6-month-old female mice. The most abundant 22 families identified in the gut microbiota of 6-month-old female mice from the most abundance to the least abundance were Muribaculaceae, Lachnospiraceae, Bacteroidaceae, Ruminococcaceae, Desulfovibrionaceae, Erysipelotrochaceae, Helicobacteraceae, Tannerellaceae, Eggerthellaceae, Rickenellaceae, Prevotellaceae, Family_XIII, Peptococcaceae, Clostridiales_vadinBB60_group, Lactobacillaceae, Cyannobiaceae, Akkermansiacceae, Alteromonadaceae, Deferribacteraceae, Lachnospiraceae_Ambiguous_taxa, uncultured_bacterium, and Staphylococcaceae (Figure 1A). Both α diversity (Figure 1B, *p* = 0.515) and β diversity (Figure 1C, *p* = 0.112) did not show any significant changes. Kruskal–Wallis test only showed significant changes on Alteromonadaceae, but not on any other families. Post hoc Games–Howell’s test further showed that Alteromonadaceae was more abundant in iTat-SA mice than WT-SA mice and iTat-CA mice (Figure 2). These results showed that in 6-month-old female mice Tat expression was associated with more Alteromonadaceae in gut, which was reversed by cocaine exposure.

**Figure 3:**
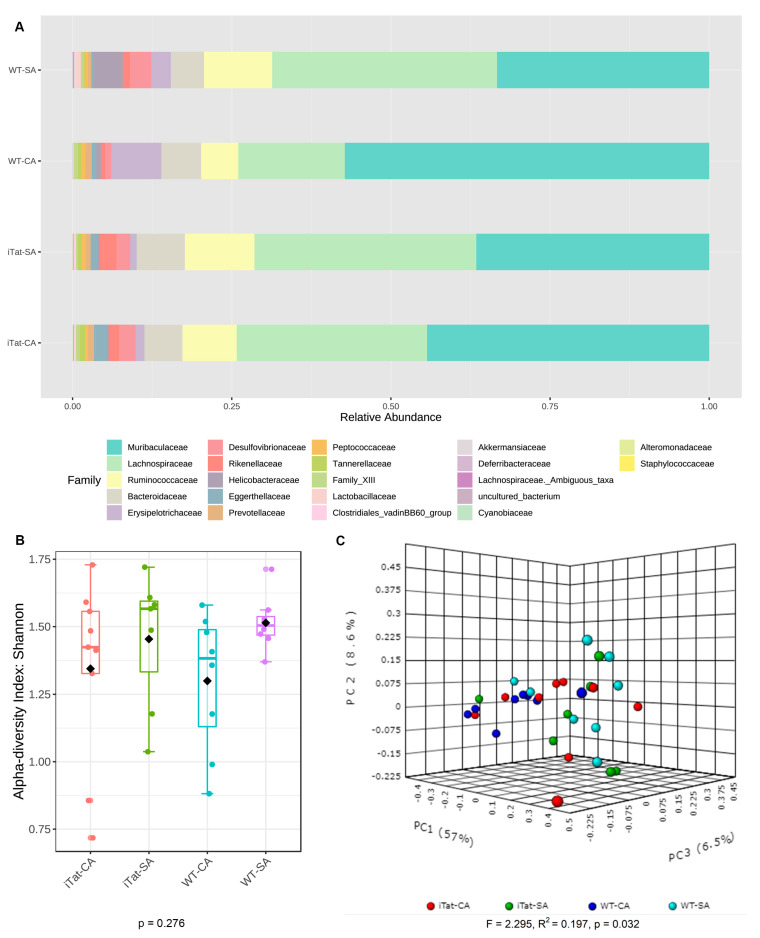
Effects of Tat expression and cocaine exposure on microbial abundance and diversity in the gut of 6-month-old male mice. (**A**). Relative abundance (%) of top 22 families of gut microbiota in four experimental groups WT-SA, WT-CA. iTat-SA, and iTat-CA were presented as a stacked bar plot. (**B**). Shannon index was calculated to determine α-diversity of the gut microbiota in four experimental groups: WT-SA, WT-CA. iTat-SA, and iTat-CA, presented as a box plot, and analyzed using the Kruskal–Wallis test. The error bars were standard derivation (SD). (**C**). Bray–Curtis dissimilarity was calculated to determine β-diversity, presented as a PCoA plot, and analyzed using PERMANOVA. The percentage variations in the plotted principal component were indicated on the axes.

**Figure 4:**
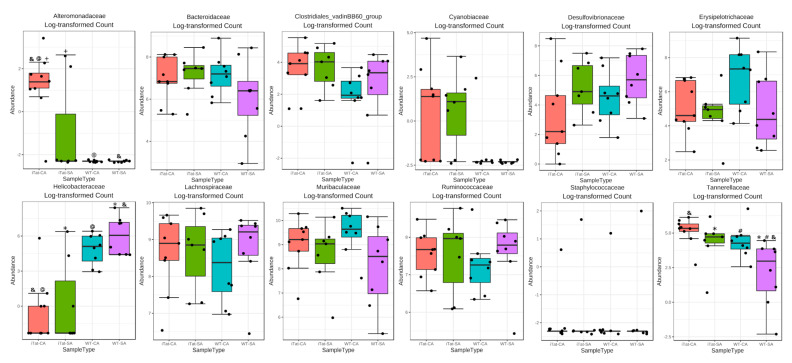
Effects of Tat expression and cocaine exposure on microbial abundance by family in the gut of 6-month male mice. Twelve families which were affected in either group were presented. The data were analyzed using Kruskal–Wallis test, followed by post hoc Games–Howell’s test. The families which were affected by Tat expression and/or cocaine exposure were marked in red. The error bars were SD, and “*”, “#”, “&”, “@” or “+” denoted *p* < 0.05.

Then, we determined the effects of Tat and cocaine on the gut microbiota of 6-month-old male mice. The most abundant 22 families identified in the gut microbiota of 6-month-old male mice were the same as these in 6-month-old female mice, but in a different order from the most abundance to the least abundance: Muribaculaceae, Lachnospiraceae, Ruminococcaceae, Bacteroidaceae, Erysipelotrochaceae, Desulfovibrionaceae, Rickenellaceae, Helicobacteraceae, Eggerthellaceae, Prevotellaceae, Peptococcaceae, Tannerellaceae, Family_XIII, Lactobacillaceae, Clostridiales_vadinBB60_group, Akkermansiacceae, Deferribacteraceae, Lachnospiraceae_Ambiguous_taxa, uncultured_bacterium, Cyannobiaceae, Alteromonadaceae, and Staphylococcaceae (Figure 3A). α diversity had no significant differences (Figure 3B, *p* = 0.276), while β diversity showed significant differences (Figure 3C, *p* = 0.032). Kruskal–Wallis test showed significant changes on Alteromonadaceae, Helicobacteraceae and Tannerellaceae. Post hoc Games–Howell’s test showed that Alteromonadaceae was more abundant in iTat-CA mice than iTat-SA, WT-CA, and WT-SA mice although Helicobacteraceae was less abundant in iTat-CA and iTat-SA mice than WT-SA mice (Figure 4). In addition, Post hoc Games–Howell’s test also showed that Tannerellaceae was more abundant in iTat-CA, iTat-SA, and WT-CA mice than WT-SA mice. These results showed that in 6-month male mice Tat expression alone, or cocaine exposure alone did not alter the abundance of Alteromonadaceae, but simultaneous Tat expression and cocaine exposure led to significant increases of the abundance of Alteromonadaceae. These results also showed that Tat expression alone significantly decreased abundance of Helicobacteraceae, and that Tat expression alone or cocaine alone had more abundant Tannerellaceae but simultaneous Tat expression and cocaine exposure significantly increased abundance of Tannerellaceae.

**Figure 5:**
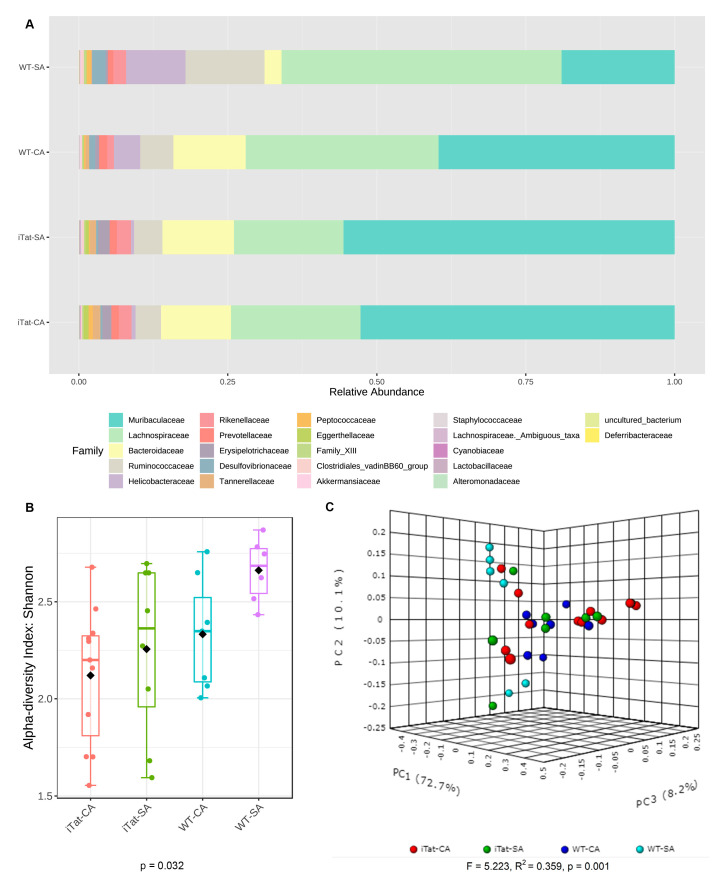
Effects of Tat expression and cocaine exposure on microbial abundance and diversity in the gut of 12-month female mice. (**A**). Relative abundance (%) of top 22 families of gut microbiota in four experimental groups WT-SA, WT-CA. iTat-SA, and iTat-CA were presented as a stacked bar plot. (**B**). Shannon index was calculated to determine α-diversity of the gut microbiota in four experimental groups WT-SA, WT-CA. iTat-SA, and iTat-CA, presented as a box plot, and analyzed using the Kruskal–Wallis test. The error bars were standard derivation (SD). (**C**). Bray–Curtis dissimilarity was calculated to determine β-diversity, presented as a PCoA plot, and analyzed using PERMANOVA. The percentage variations in the plotted principal component were indicated on the axes.

**Figure 6:**
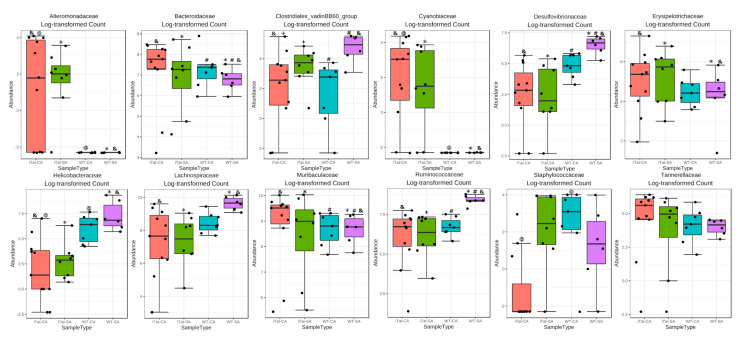
Effects of Tat expression and cocaine exposure on microbial abundance by family in the gut of 12-month female mice. Twelve families which were affected in either group were presented. The data were analyzed using Kruskal–Wallis test, followed by post hoc Games–Howell’s test. The families which were affected by Tat expression and/or cocaine exposure were marked in red. The error bars were SD, and “*”, “#”, “&”, “@” or “+” denoted *p* < 0.05.

We next determined the effects of Tat and cocaine on the gut microbiota of 12-month-old female mice. The most abundant 22 families identified in the gut microbiota of 12-month-old female mice remained the same, but in a different order from the most abundance to the least abundance: Muribaculaceae, Lachnospiraceae, Bacteroidaceae, Ruminococcaceae, Helicobacteraceae, Rickenellaceae, Prevotellaceae, Erysipelotrichaceae, Desulfovibrionaceae, Tannerellaceae, Peptococcaceae, Eggerthellaceae, Family_XIII, Clostridiales_vadinBB60_group, Akkermansiacceae, Staphylococcaceae, Lachnospiraceae_Ambiguous_taxa, Cyanobiaceae, Lactobacillaceae, Alteromonadaceae, uncultured_bacterium, and Deferribacteraceae (Figure 5A). Both α diversity (Figure 5B, *p* = 0.032) and β diversity (Figure 5C, *p* = 0.001) showed significant changes. Kruskal–Wallis test showed significant changes on Alteromonadaceae, Bacteroidaceae, Clostridiales_vadinBB60_group, Cyanobiaceae, Desulfovibrionaceae, Erysipelotrichaceae, Helicobacteraceae, Lachnospiraceae, Muribaculaceae, Ruminococcaceae, and Staphylococcaceae. Post hoc Games–Howell’s test showed that Alteromonadaceae was more abundant in iTat-CA and iTat-SA mice than WT-SA mice, that Bacteroidaceae was more abundant in iTat-CA, iTat-SA, and WT-CA mice than WT-SA mice, that Clostridiales_vadinBB60_group was less abundant in iTat-CA and WT-CA than WT-SA mice, that Cyanobiaceae was more abundant in iTat-CA and iTat-SA mice than WT-SA mice, that Desulfovibrionaceae was less abundant in iTat-CA, iTat-SA, and WT-CA mice than WT-SA mice, that Erysipelotrichaceae was more abundant in iTat-CA and iTat-SA mice than WT-SA mice, that Helicobacteraceae was less abundant in iTat-CA and iTat-SA mice than WT-SA mice, that Lachnospiraceae was less abundant in iTat-CA and iTat-SA mice than WT-SA mice, that Muribaculaceae was more abundant in iTat-CA, iTat-SA, and WT-CA mice than WT-SA mice, that Ruminococcaceae was less abundant in iTat-CA, iTat-SA, and WT-CA mice than WT-SA mice, and that Staphylococcaceae was less abundant in iTat-CA mice than WT-CA mice (Figure 6). These results showed that in 12-month-old female mice Tat expression increased the abundance of Alteromonadaceae, Bacteroidaceae, Cyanobiaceae, Erysipelotrichaceae, and Muribaculaceae and decreased the abundance of Desulfovibrionaceae, Helicobacteraceae, Lachnospiraceae, and Ruminococcaceae. These results showed that cocaine exposure alone increased the abundance of both Bacteroidaceae and Muribaculaceae and decreased the abundance of Clostridiales_vadinBB60_group, Desulfovibrionaceae’s and Ruminococcaceae.

**Figure 7:**
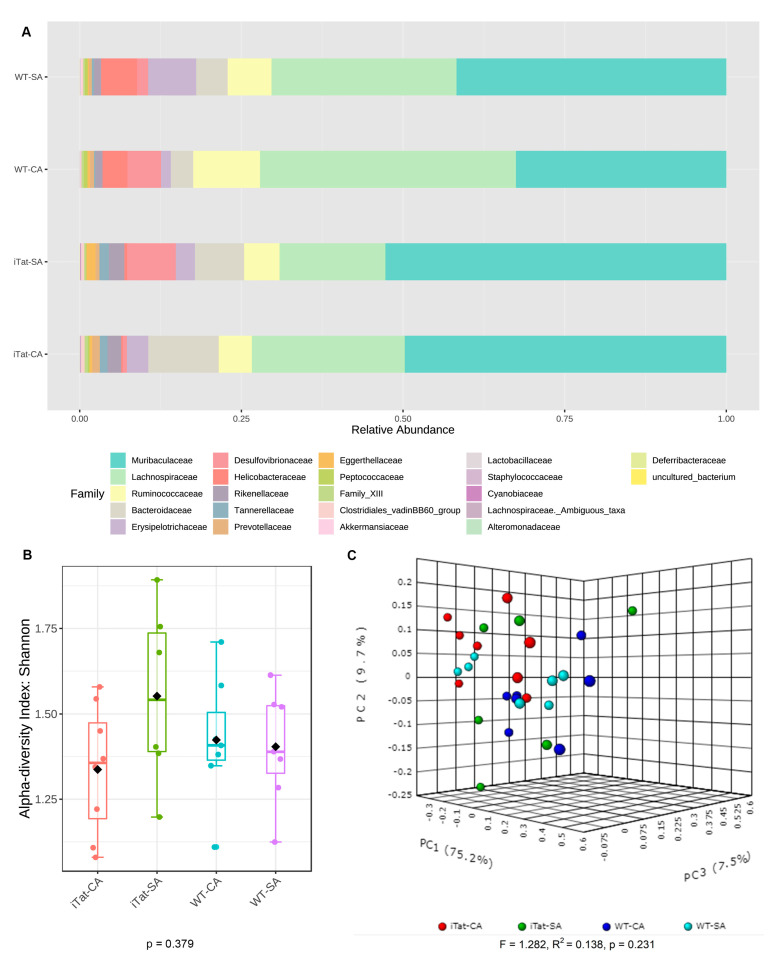
Effects of Tat expression and cocaine exposure on microbial abundance and diversity in the gut of 12-month male mice. (**A**). Relative abundance (%) of top 22 families of gut microbiota in four experimental groups, WT-SA, WT-CA, iTat-SA, and iTat-CA, were presented as a stacked bar plot. (**B**). Shannon index was calculated to determine α-diversity of gut microbiota in four experimental groups WT-SA, WT-CA. iTat-SA, and iTat-CA, presented as a box plot, and analyzed using the Kruskal–Wallis test. The error bars were standard derivation (SD). (**C**). Bray–Curtis dissimilarity was calculated to determine β-diversity, presented as a PCoA plot, and analyzed using PERMANOVA. The percentage variations in the plotted principal component were indicated on the axes.

**Figure 8:**
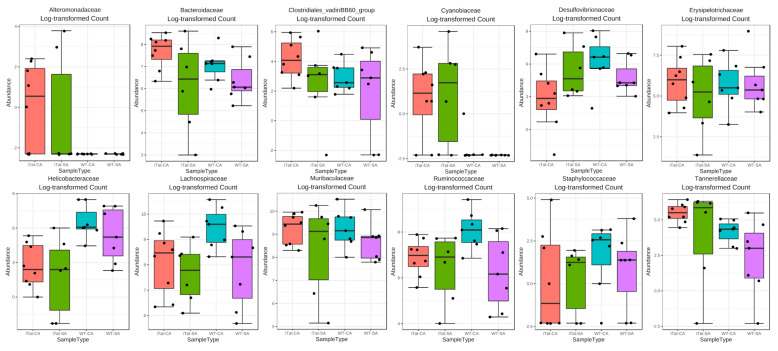
Effects of Tat expression and cocaine exposure on microbial abundance by family in the gut of 12-month male mice. Twelve families which were affected in either group were presented. The data were analyzed using Kruskal–Wallis test. The error bars were SD.

We next determined the effects of Tat and cocaine on the gut microbiota of 12-month-old male mice. The most abundant 22 families identified in the gut microbiota of 12-month-old male mice remained the same, but in a different order from the most abundance to the least abundance: Muribaculaceae, Lachnospiraceae, Ruminococcaceae, Bacteroidaceae, Erysipelotrochaceae, Desulfovibrionaceae, Helicobacteraceae, Rickenellaceae, Tannerellaceae, Prevotellaceae, Eggerthellaceae, Peptococcaceae, Family_XIII, Clostridiales_vadinBB60_group, Akkermansiacceae, Lactobacillaceae, Staphylococcaceae, Cyannobiaceae, Lachnospiraceae_Ambiguous_taxa, Alteromonadaceae, Deferribacteraceae, and uncultured_bacterium (Figure 7A). Both α diversity (Figure 7B, *p* = 0.379) and β diversity (Figure 7C, *p* = 0.231) showed no significant changes. Kruskal–Wallis test showed no significant differences in all 12 abundant families (Figure 8). These results showed that Tat expression alone, cocaine exposure alone, or simultaneous Tat expression and cocaine exposure led to no significant changes of the gut microbiota in 12-month-old male mice.

**Figure 9:**
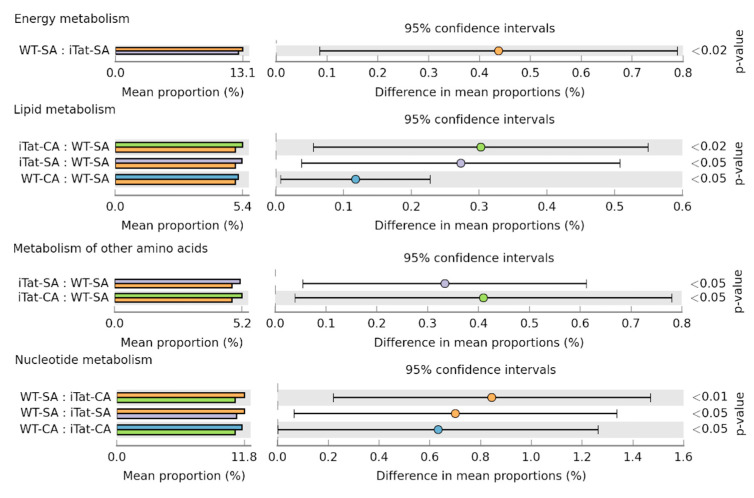
Effects of Tat expression and cocaine exposure on the metabolism in the gut of 12-month female mice. The Tax4Fun were used to estimate the changes of metabolic functions of the gut microbiota. Only significant changes of the metabolism pathways were detected in the gut of 12-month female mice and presented using Kruskal–Wallis test, followed by post hoc Games–Howell’s test.

Lastly, we analyzed all the sequence reads using the Tax4Fun to estimate the changes of microbial metabolic functions in gut for all the 16 experimental groups. In 12-month-old female mice, there was lower energy metabolism in iTat-SA mice than WT-SA mice, higher lipid metabolism in iTat-CA, iTat-SA, and WT-CA mice than WT-SA mice, higher metabolism of other amino acids in iTat-CA and iTat-SA mice than WT-SA mice, and higher nucleotide metabolism in WT-SA mice than iTat-CA and iTat-SA mice (Figure 9). The results showed that Tat expression alone decreased the energy metabolism and nucleotide metabolism, and increased the lipid metabolism and metabolism of other amino acids. In addition, cocaine exposure also increased the lipid metabolism.

## 4. Discussion

Our recent study shows that HIV Tat and cocaine interactively alter genome-wide DNA methylation and gene expression and cause neuropathological and neurocognitive impairments [63]. As an extension, we continued to characterize effects of Tat expression and cocaine exposure on the abundance, composition, and diversity of the gut microbiota from these same groups of animals in the current study. To ensure the comparability, WT mice were fed with the same Dox-containing diet as iTat mice throughout the studies. Cocaine exposure alone decreased Clostridiales_vadinBB60_group, Desulfovibrionaceae and Ruminococcaceae in the gut of 12-month-old female mice. However, Tat expression alone increased Alteromonadaceae in 6-month-old female mice and Alteromonadaceae, Bacteroidaceae, Cyanobiaceae, Erysipelotrichaceae, and Muribaculaceae in 12-month-old female mice, but decreased Desulfovibrionaceae, Helicobacteraceae, Lachnospiraceae, and Ruminococcaceae in 12-month-old female mice. These results indicate that Tat expression causes more severe dysbiosis than cocaine exposure. In contrast, cocaine exposure is associated with more diverse bacterial communities at different taxonomy levels [74,75,76]. This apparent discrepancy may be due to several factors including different sources of samples, sequencing reads processing, and use of Dox to induce Tat expression in our study. Our further analysis showed that simultaneous Tat expression and cocaine exposure increased Alteromonadaceae and Tannerellaceae in 6-month-old male mice and Bacteroidaceae in 12-month-old female mice, while Tat expression alone or cocaine exposure alone only had slight increases these families. These results suggest that Tat expression and cocaine exposure have synergistic effects in increasing this family, which is consistent with their synergistic effects on neurobehaviors and neuropathologies [63]. Interestingly, cocaine exposure appeared to attenuate Alteromonadaceae increased by Tat expression in 6-month-old female mice. Furthermore, only Tat expression but not cocaine exposure changed Alteromonadaceae, Cyanobiaceae, Erysipelotrichaceae, Helicobacteraceae, and Lachnospiraceae in 12-month female mice. Taken together, these results indicate that Tat expression and cocaine exposure exhibit different effects on different families of gut microbiota.

Aging is known to be associated with changes of the gut microbiota [77,78]. Thus, we determined the changes of bacterial communities in both 6-month-old and 12-month-old mice. Our results showed that only 1~3 families were affected by Tat expression or cocaine exposure in 6-month-old mice while 11 families were changed in 12-month-old female mice, supporting the notion that aging plays an important role in gut microbiota symbiosis. Lifestyle-induced changes of hormone or immune function may also contribute to alterations in gut microbiota, the underlying mechanism of microbiota alterations with aging is not completely clear. Our results, together with other studies, suggest that the changes of hormone or immune function induced by aging itself may play more important role in these changes [77]. At the same time, we also determined the effects of sex on gut microbiota. In 6-month-old mice, only Alteromonadaceae was affected in both female and male mice, and Helicobacteraceae and Tannerellaceae were also changed in male mice. Interestingly, completely different from 6-month-old mice, six families were altered in 12-month-old female mice, but no changes were found in 12-month-old male animals. These results indicate that sex plays an important role in gut microbiota dysbiosis. Consistent with our findings is another study in which gut microbiota shows significant differences between female and male [79]. Although male hormone can also change gut microbiota composition, most of studies focus on female hormone and the estrogen–gut microbiome axis; the concept was proposed that crosstalk exists between gut microbiota and estrogen and, in other words, gut microbiota also influence estrogen level [80]. Female is found to be more sensitive to addictive substance, including cocaine, and alterations of host microbiota affect cocaine-induced behavioral activities [81]. Therefore, the changes in gut microbiota between female and male may eventually contribute to the differences of behavioral activities between female and male through the gut–brain axis. Taken together, both age and sex are important factors in determining the gut microbiota composition.

The normal gut microbiota primarily consists of four phyla Bacillota (also known as Firmicutes), Bacteroidota, Actinomycetota, and Verrucomicrobiota and mainly functions to provide nutrients, protection against pathogens, and immune response [82,83,84]. The families that were altered by Tat expression, cocaine exposure, or simultaneous Tat expression and cocaine exposure from the current study were Alteromonadaceae in 6-month-old female/male mice and Bacteroidaceae, Desulfovibrionaceae, Erysipelotrichaceae, Lachnospiraceae, Muribaculaceae, and Ruminococcaceae in 12-month-old female mice. These families belong to the phyla Bacillota (Ruminococcaceae, Lachnospiraceae, and Erysipelotrichaceae), Bacteroidota (Muribaculaceae and Bacteroidaceae), and Thermodesulfobacteriota (Desulfovibrionaceae), and Pseudomonadota (Alteromonadaceae). Lack of the phyla Actinomycetota, and Verrucomicrobiota in the gut microbiota from this study is likely due to use of doxycycline in all the mice [82]. In this study, we also found Tat expression or cocaine exposure resulted in metabolic abnormalities which are common in HIV-infected patients. Consistent with our findings, Tat expression results in a decrease in cellular energy metabolism by deregulating intracellular calcium homeostasis and disrupting mitochondrial function [85], and cocaine exposure increases lipid metabolism [86]. However, the relationship between changes of these families and changes of metabolic pathways in the gut of 12-month-old female mice remains to be determined.

An increasing number of recent studies support the bidirectional gut–brain axis hypothesis that the brain can alter microbial composition in gut by autonomic nervous system, and that gut microbiota, in turn, can regulate the brain function by endocrine and neurocrine pathways [87]. In this study, we chose iTat mice as a surrogate HAND model to explore possible bidirectional interactions between HAND and gut microbiota. Tat protein is expressed in astrocytes, secreted from these cells, and taken up by other cells such as neurons [88,89,90,91,92,93]. Tat may be transported from the brain to the peripheral tissues/organs such as gut and lead to change of the gut microbiota, which in turn contributes to HAND. There is another possibility that Tat is induced in the glial fibrillary acid protein-positive glial cells, which directly regulate proinflammatory response, microbiota composition, and epithelial barrier integrity in gut and then contribute to neuroinflammation and changes of neurobehaviors [94,95]. On the other hand, cocaine was given to mice through the i.p. route in this study. Thus, cocaine’s effects on the gut microbiota could be direct or indirect. Nevertheless, further studies are needed to understand the underlying mechanisms of how Tat and cocaine affect the gut microbiota and whether these mechanisms can be explored for development of HAND therapeutics. In conclusion, the findings from the current study show that Tat expression and cocaine exposure lead to most changes of microbiota in adult female mice.

## Data Availability

The sequencing data sets are available at NCBI under the project #PRJNA942335 and submission #SUB12936855 via the link https://dataview.ncbi.nlm.nih.gov/object/PRJNA942335?reviewer=fcqrm7jfa5ij102nmkle8bssa4 (accessed on 16 March 2023).
